# The opioid receptor pharmacology of GSK1521498 compared to other ligands with differential effects on compulsive reward-related behaviours

**DOI:** 10.1007/s00213-014-3666-3

**Published:** 2014-06-29

**Authors:** Eamonn Kelly, Stuart J. Mundell, Anna Sava, Adelheid L. Roth, Antonio Felici, Kay Maltby, Pradeep J. Nathan, Edward T. Bullmore, Graeme Henderson

**Affiliations:** 1 School of Physiology and Pharmacology, University of Bristol, Bristol, BS8 1TD UK; 2 Aptuit Centre for Drug Discovery & Development, Aptuit Srl., Verona, Italy; 3 Medicines Discovery and Development, GlaxoSmithKline, Clinical Unit Cambridge, Cambridge, UK; 4 Brain Mapping Unit, Department of Psychiatry, University of Cambridge, Cambridge, UK

**Keywords:** μ-Opioid receptor, Inverse agonism, GSK1521498

## Abstract

**Rationale:**

The novel opioid receptor antagonist, GSK1421498, has been shown to attenuate reward-driven compulsive behaviours, such as stimulant drug seeking or binge eating, in animals and humans. Here, we report new data on the receptor pharmacology of GSK121498, in comparison to naltrexone, naloxone, 6-β-naltrexol and nalmefene.

**Objectives:**

To determine whether the novel opioid antagonist, GSK1521498, is an orthosteric or allosteric antagonist at the μ opioid receptor (MOPr) and whether it has neutral antagonist or inverse agonist properties.

**Methods:**

A combination of radioligand binding assays and [^35^S]GTPγS binding assays was employed.

**Results:**

GSK1521498 completely displaced [^3^H]naloxone binding to MOPr and did not alter the rate of [^3^H]naloxone dissociation from MOPr observations compatible with it binding to the orthosteric site on MOPr. GSK1521498 exhibited inverse agonism when MOPr was overexpressed but not when the level of MOPr expression was low. In parallel studies under conditions of high receptor expression density, naloxone, naltrexone, 6-β-naltrexol and nalmefene exhibited partial agonism, not inverse agonism as has been reported previously for naloxone and naltrexone. In brain tissue from mice receiving a prolonged morphine pre-treatment, GSK1521498 exhibited slight inverse agonism.

**Conclusions:**

Differences between GSK1521498 and naltrexone in their effects on compulsive reward seeking are arguably linked to the more selective and complete MOPr antagonism of GSK1521498 versus the partial MOPr agonism of naltrexone. GSK1521498 is also pharmacologically differentiated by its inverse agonist efficacy at high levels of MOPr expression, but this may be less likely to contribute to behavioural differentiation at patho-physiological levels of expression.

## Introduction

GSK1521498 (N-{[3,5-difluoro-3′-(1H-1,2,4-triazol-3-yl)-4-biphenylyl]methyl}-2,3-dihydro-1H-inden-2-amine phosphate (1:1)) is a novel opioid receptor antagonist under clinical development for disorders of compulsive consumption (Ignar et al. 2011; Nathan et al. 2012a, b). Behavioural studies in rodent models of compulsive consumption have demonstrated that GSK1521498 can significantly, and dose dependently, attenuate compulsive searching for cocaine, heroin or chocolate rewards and compulsive consumption of heroin or food rewards (Giuliano et al. 2012, 2013). GSK1521498 could be behaviourally differentiated from naltrexone in these experiments: GSK1521498 in the dose range 0.1–3 mg/kg had greater effects than equivalent doses of naltrexone on cocaine seeking, heroin seeking and heroin consumption. For example, GSK1421598 3 mg/kg was associated with a 2.5-fold greater reduction in cocaine seeking and a 5.5-fold greater reduction in heroin seeking, compared to naltrexone 3 mg/kg (Giuliano et al. 2013).

Here, we report a detailed characterization of the receptor pharmacology of GSK1521498 compared to naltrexone, and its principal metabolite 6-β-naltrexol, in cell lines and in brain tissue from morphine-treated animals. We hypothesized that the behavioural differences between GSK1521498 and naltrexone observed previously could be related to pharmacological differences in μ-opioid receptor (MOPr) selectivity or efficacy.

In an initial study of its receptor pharmacology, GSK1521498 was reported to be selective for recombinant human MOPr over κ (KOPr) and δ (DOPr) (Ignar et al. 2011). Furthermore, in cell membranes from CHO-Gam E1A cells virally expressing high levels of recombinant receptor, GSK1521498 exhibited inverse agonism, at MOPr, KOPr and DOPr. In contrast, the classical opioid antagonist naltrexone demonstrated neutral antagonist/partial agonist activity at MOPr and KOPr and neutral antagonist activity at DOPr (Ignar et al. 2011).

Under certain experimental conditions, MOPrs have been reported to exhibit constitutive activity which can be inhibited by some opioid antagonists indicating that they may also have inverse agonist activity (Burford et al. 2000; Liu et al. 2001; Wang et al. 2001, 2004, 2007). For example, β-chlornaltrexamine (βCNA) was reported to reduce the basal level of GTPγS binding in membranes prepared from either mouse brain or cell lines expressing recombinant MOPr (Wang et al. 2004). While the inverse agonism induced by βCNA was apparent in brain membranes prepared from control mice, it was enhanced in membranes prepared from mice pretreated with morphine for 1 or 3 days when constitutive activity of MOPr was reported to be increased. Furthermore, while naltrexone and naloxone exhibited neutral antagonist activity in control membrane preparations, these drugs also exhibited inverse agonist effects in membranes from morphine-pre-treated cells (Wang et al. 2001, 2007) or membranes prepared from morphine-treated mice brains (Wang et al. 2004). The principal (active) metabolite of naltrexone, 6-β-naltrexol, was reported to act as a neutral antagonist irrespective of the conditions of the experiment (Wang et al. 2001, 2004, 2007).

Other studies, however, have failed to observe inverse agonist activity with MOPr antagonists and have observed either neutral antagonism or weak partial agonism (for review see Connor and Traynor 2010). For example, in different studies, naloxone has been reported to possess positive (Burford et al. 2000; Liu and Prather 2001; Wang et al. 2007), neutral (Brillet et al. 2003; Divin et al. 2009) or negative intrinsic activity (Liu and Prather 2001; Sally et al. 2010; Wang et al. 2004, 2007). The issue of whether MOPrs exhibit constitutive activity and thus whether some antagonists exhibit inverse agonism is still controversial (Connor and Traynor 2010; Williams et al. 2013).

The objective of the present study was to characterize the effects of GSK1521498 at opioid receptors in vitro and to compare them with several established opioid antagonists that have previously been reported to have neutral antagonist or inverse agonist activity: naltrexone, 6-β-naltrexol, nalmefene, βCNA. We confirmed that GSK1521498 showed selectivity for MOPr over the other opioid receptors and have additionally demonstrated that it binds to the orthosteric binding site on MOPr. We demonstrated that GSK1521498 could more completely block MOPr transmission than naltrexone, which had partial agonist efficacy. In contrast, we found further evidence that GSK1521498 had some inverse agonist activity, but this was observed using highly expressed recombinant MOPr, or endogenous MOPr from mice pre-treated with the highest dose of morphine, but not endogenous MOPr from control mice or mice pre-treated with lower doses of morphine. We were unable to observe inverse agonist activity using βCNA, nalmefene, naltrexone or 6-β-naltrexol under any assay conditions.

## Methods and materials

### Preparation of membranes

(i) MOPr-, DOPr-, KOPr- and ORL1 (NOPr-) Chinese hamster ovary (CHO) cells
  CHO cells stably expressing human MOPr, DOPr, KOPr and NOPr, respectively, were grown to ~90 % confluency, washed with PBS prior to being detached with Versene or trypsin and collected by centrifugation (330 g, 5 min, 4 °C). The cell pellets were rinsed once in PBS and homogenized in ten volumes (*w*/*v*) of membrane preparation buffer (50 mM HEPES, 0.1 mM leupeptin, 1 mM EDTA, 0.1 mM Pefabloc and pepstatin A pH = 7.4) using an Ultra-Turrax homogenizer (2 × 15-s bursts). The homogenates were then centrifuged at 48,000 × *g* for 20 min at 4 °C, and the pellets were washed once more in membrane preparation buffer and re-centrifuged as before. The final pellets were resuspended in five volumes of membrane preparation buffer and frozen at −80 °C until use. Protein concentration was determined by the Bio-Rad Protein assay using bovine serum albumin (BSA) as the standard.
(ii) MOPr-human embryonic kidney (HEK) 293 cells
  HEK293 cells stably expressing human MOPr (approximately 1,600 fmol/mg protein) were grown to ~90 % confluency then gently washed twice with 2-ml ice-cold hypotonic lifting buffer (10 mM HEPES, 0.9 % *w*/*v* NaCl, 0.2 % *w*/*v* EDTA, pH 7.4). Cells were then removed from the bottom of the dish using an Iwaki cell scraper and suspended in 2 ml of ice-cold lifting buffer. The cells were pelleted by centrifugation (377 × *g*, 10 min, 4 °C) before discarding the supernatant and resuspending in 2 ml of ice-cold wash buffer 1 (10 mM HEPES, 10 mM EDTA, pH 7.4). The centrifugation step was then repeated and the wash cycle repeated twice more. The washed cell suspension was then homogenised using 4 × 5-s bursts with an Ultra-Turrax homogeniser. The homogenate was then centrifuged (48,000 × *g*, 30 min, 4 °C) before the supernatant was discarded and pellet was resuspended in 2 ml of ice-cold wash buffer 1. The centrifugation was then repeated, before resuspension of the pellet in 3 ml of ice-cold wash buffer 2 (10 mM HEPES, 0.1 mM EDTA, pH 7.4) at protein concentrations of 2–5 mg/ml. Aliquots of membrane protein were stored at −80 °C until use.
(iii) Mouse brain
  All animal experiments were carried out in accordance with the UK Animals (Scientific Procedures) Act, 1986, and associated guidelines. Male CD1 mice (25–30 g) were obtained from Banting and Kingman (UK) and housed at the University of Bristol’s animal facility. They had access to food and water ad libitum. Four groups of mice were used.
  1. Naive mice—untreated
  2. Acute morphine-pretreated mice received 100 mg/kg morphine subcutaneous (sc) 4 h before sacrifice
  3. Moderate morphine-pretreated mice received sc injections of morphine at 8-h intervals for 3 days (day 1—3 × 30 mg/kg, day 2—3 × 60 mg/kg, day 3—3 × 100 mg/kg). Animals were sacrificed 12 h later on day 4.
  4. Severe morphine-pretreated mice were implanted sc under isoflurane anaesthesia with a morphine pellet containing 75 mg morphine. Animals were sacrificed 3 days after pellet implantation. It has been estimated that this treatment results in brain morphine levels that peak around 600 ng/g tissue and remain above 200 ng/g tissue over the period of administration (Patrick et al. 1975)

The categorization of morphine pre-treatment is as described by Raehal et al. (2005).

Brain membranes were prepared as described by Wang et al. (2004). Animals were killed by cervical dislocation and their brains removed, placed in Eppendorf tubes and frozen in liquid nitrogen. The tubes were then stored at −80 °C until used. To prepare membranes, brains were homogenized (4 × 5-s bursts using an Ultra-Turrax homogeniser) in buffer containing 50 mM Tris-HCl, pH 7.5, 100 mM NaCl, 1 mM EDTA, 1 mM dithiothreitol (DTT), 1 mM MgCl_2_ and 0.25 M sucrose at 4 °C and then centrifuged at 27,000 × *g* for 10 min (4 °C). The resulting pellet was resuspended in homogenizing buffer and centrifuged twice more (as described above), before the final pellet was resuspended in homogenizing buffer and stored in aliquots at −80 °C. The protein concentration of the aliquots was determined to be naïve 2.41 mg/ml, morphine acute 2.35 mg/ml, morphine moderate 2.22 mg/ml and morphine severe 2.21 mg/ml.

### [^35^S]GTPγS binding assay

[^35^S]GTPγS binding studies in CHO cell membranes from MOPr overexpressing cells were performed in 384-well format using scintillation proximity assays (SPAs). MOPr, DOPr, KOPr and NOP membranes were diluted to 10, 20, 30 and 2 μg/ml, respectively, in assay buffer (20 mM HEPES, 10 mM MgCl_2_, 100 mM NaCl, pH 7.4) supplemented with 5 μM GDP, 30 μg/ml saponin, 0.01 % Pluronic F1275, 5 mg/ml wheat germ agglutinin-polystyrene imaging beads (PerkinElmer) and 0.5 nM [^35^S]GTPγS (1,250 Ci/mmol). The reaction mixtures were incubated for 2 h at 25 °C with different concentrations of test compound or vehicle (DMSO) in the absence (agonist mode) or presence (antagonist mode) of a sub-maximal concentration of agonist (Met-Enk, dynorphin A and nociceptin for MOPr/DOPr, KOPr and NOPr, respectively). The final assay volume was 20 μl for MOPr and NOP and 40 μl for DOPr and KOPr. Basal [^35^S]GTPγS binding was determined in the absence of compounds. Bound [^35^S]GTPγS was determined by scintillation counting on a ViewLux microplate imager (Wallac 1430, PerkinElmer).

To study potential inverse agonism at MOPr in mouse brain membranes and CHO cells expressing low levels of MOPr, we used conditions identical to those of Wang et al. (2004), using an assay buffer containing 50 mM Tris-HCl pH 7.5, 100 mM NaCl, 4 mM MgCl_2_, 1 mM DTT, 10 μM GDP, 1 mM EDTA and 0.1 % BSA. Brain membranes (10 μg/tube) were incubated with assay buffer as well as drug and 0.1 nM [^35^S]GTPγS (1,250 Ci/mmol) at 30 °C for 30 min before rapid filtration on a Brandel Cell Harvester using Whatman GF/B filters and scintillation counting.

### Radioligand binding assay

Membranes were prepared from MOPr-HEK 293 cells as described above. For competition binding experiments, competing ligands were prepared in increasing concentrations in HBSS/20 mM HEPES/pH 7.4, in LP4 tubes containing 10 μg of protein per well. Then, 4 nM [^3^H]naloxone was added to each tube, and binding reactions were left to incubate at 22 °C for 2 h with agitation. In parallel samples, non-specific binding was determined with 1 μM etorphine. Both total binding and non-specific binding curves were performed in duplicate. Membranes were then harvested onto filter paper discs moistened with ice-cold wash buffer: HEPES 20 mM, pH7.4. Each disc of filter paper was placed in a scintillation vial and 3-ml Emulsifier-Safe scintillation fluid added. Samples were left for 3 h before reading in a scintillation counter. For competition dissociation binding experiments, 10 μg of protein plus 4 nM [^3^H]naloxone was added to each tube, and binding reactions were left to incubate at 22 °C for 2 h with agitation. Then, 3 ml of quenching solution containing unlabelled naloxone (1 μM) to prevent rebinding of [^3^H]naloxone to the orthosteric site ± either GSK1521498 (1 μM), 6-β-naltrexol (1 μM) or naltrexone (1 μM) was added and the incubation continued for various times from 0 s to 15 min. In parallel samples, non-specific binding was determined with 1 μM etorphine. Membranes were then harvested and radioactivity bound measured as described above for competition binding experiments.

### Drugs and reagents

Guanosine 5′-*O*-(3-[^35^S]thio)triphosphate ([^35^S]GTPγS; 1,250 Ci/mmol) and [^3^H]naloxone (1 mCi/ml) were from PerkinElmer. Drugs and reagents were obtained from the following sources: β-chlornaltrexamine dihydrochloride (β-CNA), methionine enkephalin acetate salt hydrate (Met-Enk), dynorphin A (porcine), naltrexone, 6-β-naltrexol, nalmefene and materials for membrane preparation (all from Sigma); etorphine hydrochloride and morphine base pellets (from RTI); morphine hydrochloride (from Macfarlan Smith); nociceptin and naloxone hydrochloride (from Tocris).

### Data analysis and statistics

Concentration-response curves and ligand-displacement curves were determined by fitting data from individual experiments to sigmoidal concentration-response curves with variable slope in Graphpad Prism 5.0, with fpKi (pKi measured in a functional assay) and pKi (measured in a radioligand binding assay) and S.E.M. calculated using individual values from each experiment. The dissociation of [^3^H]naloxone in the absence and presence of other ligands was fitted to a single exponential decay in Graphpad Prism 4.0. Data were analyzed using a Student’s *t* test, ANOVA or one-way ANOVA with Bonferroni post-test as appropriate.

## Results

### Opioid receptor selectivity

**Fig. 1:**
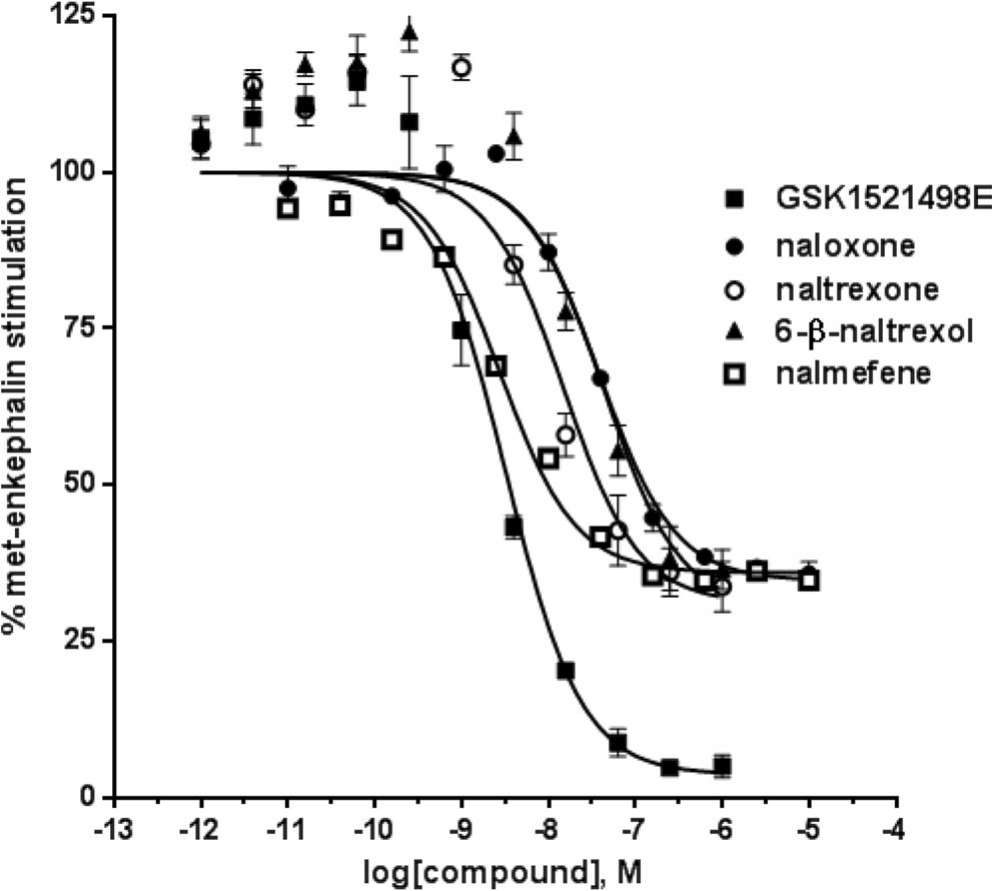
Inhibition of Met-Enk-stimulated [^35^S]GTPγS binding to membranes prepared from CHO cells overexpressing MOPr. Concentration-response curve for GSK1521498 (*black square*), naloxone (*black circle*), naltrexone (*white circle*), 6β-naltrexol (*black up-pointing triangle*) and nalmefene (*white square*) for inhibiting 10-nM Met-Enk-stimulated [^35^S]GTPγS binding in CHO cells expressing MOPr. Mean data are expressed as percentage of the stimulation produced by Met-Enk and are from three experiments each performed in duplicate. For each curve, the maximum was constrained to 100 % and the slope to unity

To determine the antagonist selectivity of GSK1521498 for opioid receptors, we examined its ability to antagonize agonist-activated [^35^S]GTPγS binding in cell membranes prepared from CHO cells overexpressing either MOPr, DOPr, KOPr or NOPr and compared it to four other opioid antagonists, naloxone, naltrexone 6-β-naltrexol and nalmefene. The agonists used to stimulate each opioid receptor subtype were Met-Enk (MOPr and DOPr), dynorphin (KOPr) and nociceptin (NOPr). GSK1521498 showed antagonist activity at all four opioid receptors and was the most potent antagonist at MOPr (Fig. 1 and Table 1). It had 14-fold selectivity for MOPr over DOPr and KOPr and 100-fold selectivity for MOPr over NOPr. When administered alone, GSK1521498 did not show agonist activity at any of the opioid receptors at concentrations up to 100 μM (see Fig. 3 for MOPr; data not shown for KOPr and DOPr). It did exhibit slight inverse agonism at MOPr (see below and Fig. 3) but not at any of the other opioid receptors.

**Table 1:** Antagonist potency (fpKi values) for the antagonists at opioid receptors

	MOPr	DOPr	KOPr	NOPr
GSK1521498	9.64 ± 0.06	8.50 ± 0.03	8.48 ± 0.14	6.59 ± 0.13
Naloxone	8.68 ± 0.14	7.51 ± 0.16	7.50 ± 0.16	<5
Naltrexone	9.15 ± 0.13	8.26 ± 0.10	8.78 ± 0.07	<5
6β-Naltrexol	8.72 ± 0.11	7.71 ± 0.10	8.52 ± 0.05	<5
Nalmefene	9.62 ± 0.12	8.08 ± 0.06	–	<5

Data are presented as mean ± SEM of three experiments

In contrast, naloxone, naltrexone, 6-β-naltrexol and nalmefene did not produce complete inhibition of the agonist responses at MOPr (Fig. 1), which is compatible with their partial agonist activity in this expression system (see below and Fig. 3).

### Binding to the MOPr

Radioligand-binding studies can provide important information on how a ligand interacts with a receptor, not only the affinity of binding but also whether it binds at the orthosteric site or at an allosteric site on the receptor.

**Fig. 2:**
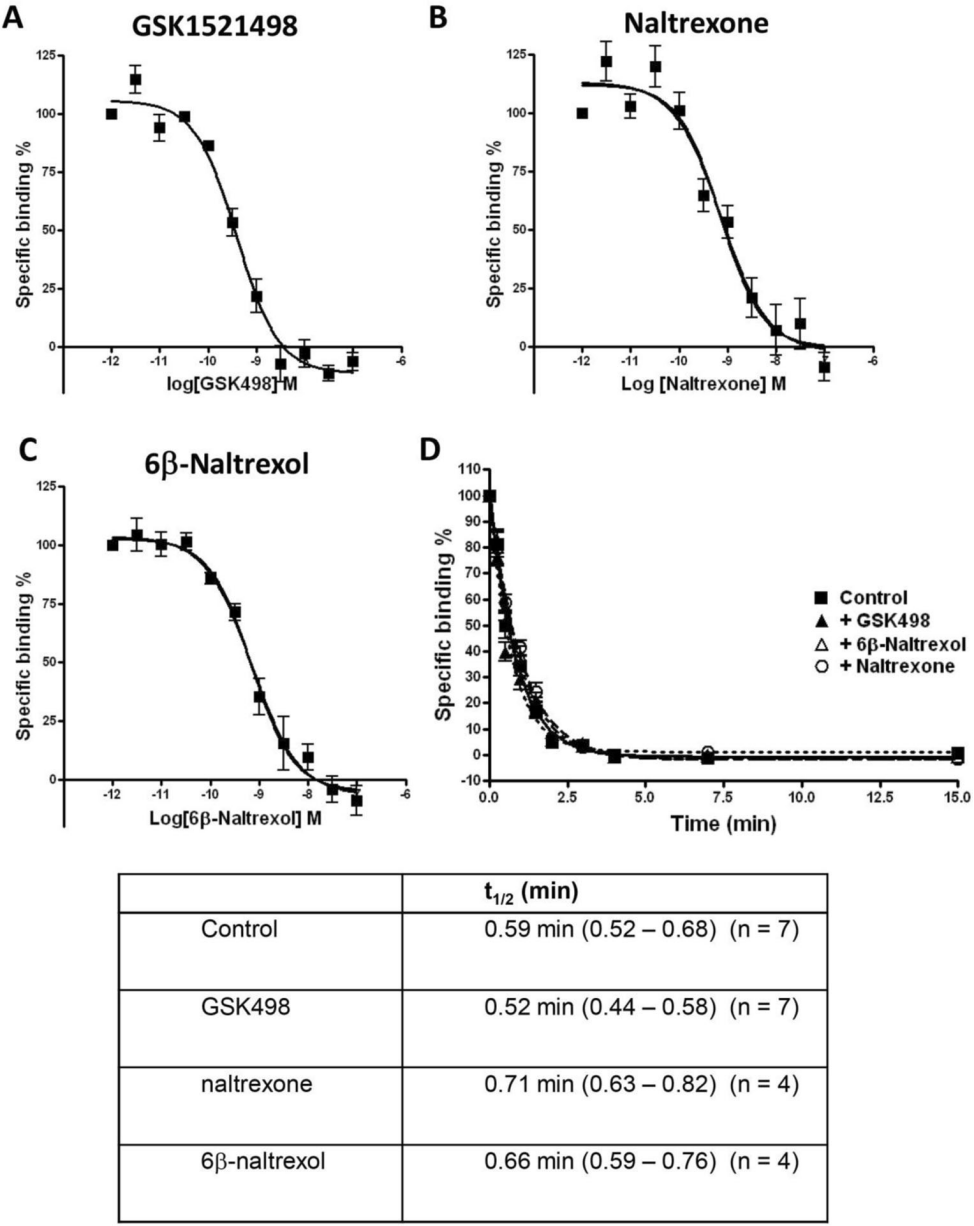
Displacement of [^3^H]naloxone binding to MOPr. **a**–**c** Competition displacement binding curves for the displacement of ^3^H-naloxone binding by GSK1521498, naltrexone, and 6-β-naltrexol to membranes prepared from HEK293 cells expressing the human MOPr. **d** Kinetics of [^3^H]naloxone dissociation from MOPr in the absence and presence of GSK1521498, naltrexone and 6-β-naltrexol (all at 1 μM). Mean t_1/2_ values for [^3^H]naloxone dissociation in the presence of each drug are given with 95 % confidence limits

Competition displacement binding curves were constructed for the displacement of ^3^H-naloxone binding by GSK1521498, naltrexone and 6-β-naltrexol from MOPrs expressed in HEK293 cells. All three compounds displaced specific ^3^H-naloxone binding in a concentration-dependent manner with the maximum displacement being 100 % for each (Fig. 2a–c). pKi values calculated using the Cheng-Prussoff equation are given in Table 2. The order of affinity of binding to human MOPr was GSK1521498 > naltrexone = 6-β-naltrexol.

**Table 2:** Affinity of binding (pKi) of MOPr antagonists to MOPr expressed in HEK293 cells

	pKi
GSK1521498	9.38 ± 0.10 (*n* = 7)
Naltrexone	9.15 ± 0.04 (*n* = 6)
6β-Naltrexol	9.12 ± 0.09 (*n* = 6)

Data are presented as mean ± SEM

To further examine whether GSK1521498 might act at an allosteric site on MOPr to alter binding at the orthosteric ligand binding site, we determined whether it altered the rate of ^3^H-naloxone dissociation from MOPrs. Non-radioactive naloxone was added to prevent rebinding of ^3^H-naloxone once it had dissociated from MOPr. The dissociation of ^3^H-naloxone from MOPr at 22 °C could be fitted by a single exponential (Fig. 2d). The t_1/2_ for dissociation was 0.59 min (95 % confidence limits 0.52–0.68; *n* = 7). In the presence of GSK1521498 (1 μM), the rate of ^3^H-naloxone dissociation was unchanged (Fig. 2d). Similarly, the rate of ^3^H-naloxone dissociation was unchanged in the presence of naltrexone (1 μM) or 6-β-naltrexol (1 μM) (Fig. 2d). These data are compatible with GSK1521498, naltrexone and 6-β-naltrexol binding to the orthosteric site on MOPr to which naloxone binds.

### Inverse agonism at MOPr

**Fig. 3:**
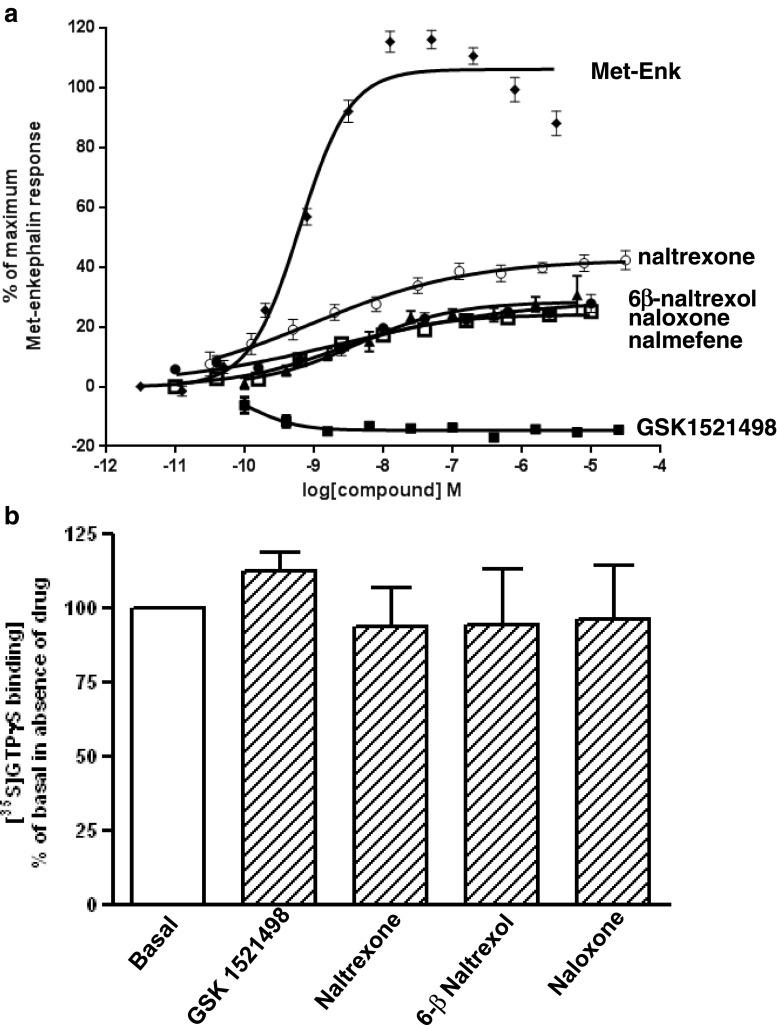
Effect of antagonists on basal [^35^S]GTPγS binding to membranes from CHO cells expressing MOPr. **a** Concentration-response curve for GSK1521498 (*black square*), naloxone (*black circle*), naltrexone (*white circle*), 6-β-naltrexol (*black up-pointing triangle*) and nalmefene (*white square*) in CHO cell membranes overexpressing MOPr. Mean data are expressed as percentage of the basal binding, determined in the absence of ligands. At concentrations greater than 0.4 nM, GSK1521498 produced a significant inhibition of basal [^35^S]GTPγS binding (*p* < 0.001, ANOVA on log-transformed raw data, with treatment and plates as independent factors). **b** Drugs were tested at a final concentration of 1 μM on membranes prepared from CHO cells expressing a low level of MOPr. Values are expressed as a percentage of the basal value in the absence of drug, which was taken as 100 % in each experiment. Values shown as means ± SEM from five separate experiments in each case. None of the drugs had a significant effect on basal [^35^S]GTPγS binding (one sample *t* test)

In GTPγS binding studies on CHO cell membranes expressing recombinant MOPrs, we observed that GSK1521498 produced a slight inhibition (up to 18 %) of basal [^35^S]GTPγS binding (Fig. 3). This would indicate some inverse agonist activity of GSK1521498. In contrast, naltrexone, naloxone, 6-β-naltrexol and nalmefene enhanced basal [^35^S]GTPγS binding (by 40, 20, 30 and 20 %, respectively) indicating partial agonist activity, not inverse agonist activity of these compounds (Fig. 3a). The partial agonist activity of these antagonists would explain why they did not completely inhibit the agonist actions of Met-Enk on MOPr shown in Fig. 1.

In these recombinant MOPr-GTPγS binding assays, we used highly overexpressed MOPrs in CHO cell membranes (Bmax 14,500 fmol/mg protein). The overexpression of MOPr is evidenced by the high potency of Met-Enk to stimulate [^35^S]GTPγS binding (EC_50_ 0.6 nM, 95 % confidence limits 0.4 to 0.8 nM): In rat brain neurons, the EC_50_ for Met-Enk activation of the K^+^ current was reported to be 1 μM (Bailey et al. 2009). We next examined whether GSK1521498 exhibited inverse agonism at MOPr in CHO cells when the level of expression was lower, at the level of endogenous MOPr expression in the brain. We used membranes from CHO cells that expressed MOPr at a level of approximately 70 fmol per milligram of protein. In these membranes, GSK1521498, naltrexone, naloxone and 6-β-naltrexol (all at 1 μM) did not modify the basal level of GTPγS binding (Fig. 3b). In contrast, DAMGO (1 μM) still stimulated [^35^S]GTPγS binding by over 50 % in these membranes (data not shown).

To further examine whether GSK1521498 has inverse agonist properties at MOPr, we examined its ability to reduce [^35^S]GTPγS binding in membranes from mouse brain. It has previously been reported that while the inverse agonist activity of β-CNA can be observed in brain membranes from naïve mice, constitutive MOPr activity is enhanced by morphine pre-treatment, and this reveals the inverse agonist activity of other antagonists such as naltrexone and naloxone (Wang et al. 2001, 2004). We compared the inverse agonist properties of GSK1521498, β-CNA, naltrexone and 6-β-naltrexol in membranes prepared from naïve mice and mice pretreated with morphine. We used three morphine pre-treatments, as described in the “Methods and materials”. These are characterized as acute, moderate and severe morphine pre-treatments (Raehal et al. 2005).

**Fig. 4:**
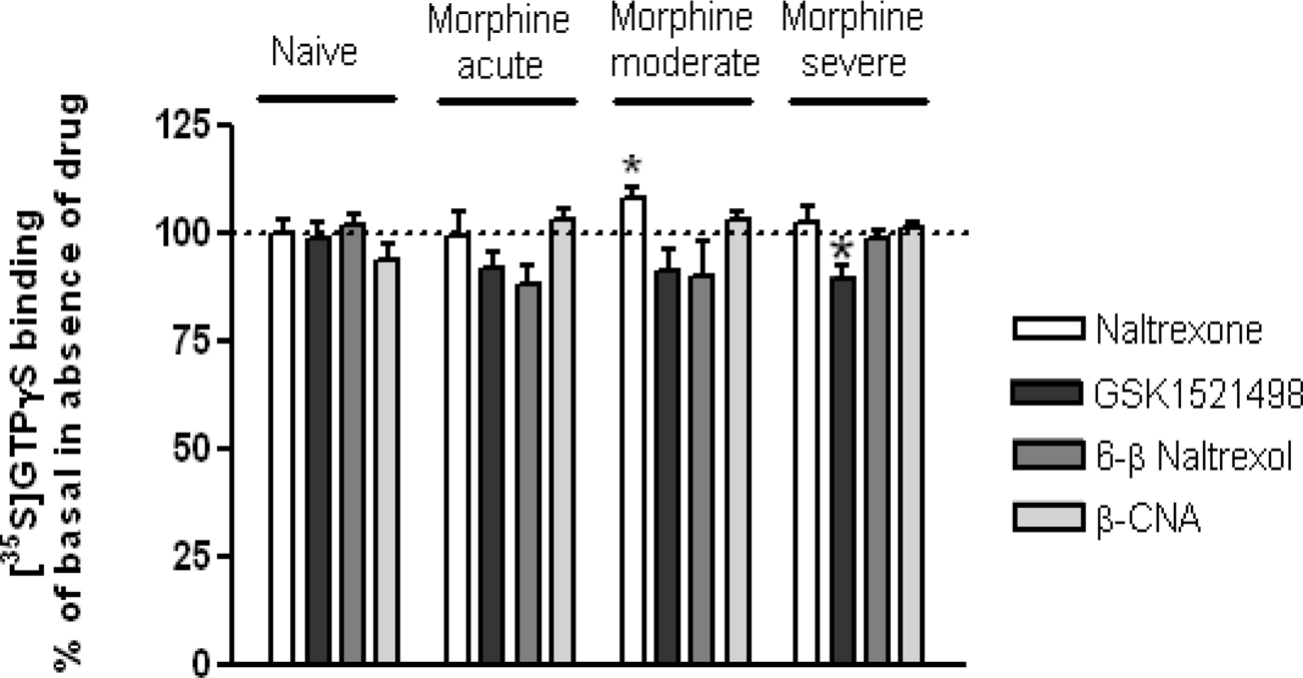
Effect of antagonists on basal [^35^S]GTPγS binding to membranes from whole mouse brain, following different morphine pre-treatments. Drugs were tested at a final concentration of 1 μM. Values are expressed as a percentage of the basal value in the absence of antagonist drug, which was taken as 100 % in each experiment. Values shown as means ± SEM from five to six separate experiments in each case, each performed in triplicate or quadruplicate.*p < 0.05 compared to 100 %, one-sample *t* test

Basal [^35^S]GTPγS binding levels were the same in brain membranes prepared from naïve mice and from mice receiving acute morphine, moderate morphine and severe morphine pre-treatments (Table 3), i.e. morphine pre-treatment did not enhance MOPr constitutive activity. Furthermore, we did not observe any inhibition of basal [^35^S]GTPγS binding by β-CNA (1 μM) or naltrexone (1 μM) in membranes from naïve or morphine-treated animals (Fig. 4). We did, paradoxically, observe a small increase in GTPγS binding with naltrexone in membranes prepared from mice receiving moderate but not acute or severe morphine pre-treatment. The lack of any inverse agonist effect of β-CNA was surprising given previous studies (Wang et al. 2001, 2004). We therefore sought to demonstrate that the sample of β-CNA used did bind to MOPr and functioned as an antagonist. β-CNA displaced specific ^3^H-naloxone binding in membranes from MOPr-HEK cells in a concentration-dependent manner and prevented DAMGO stimulation of GTPγS binding in mouse brain membranes (data not shown).

**Table 3:** Basal [^35^S]GTPγS binding to membranes of mouse brain following various morphine treatments

[^35^S]GTPγS binding			
Naive	Morphine acute	Morphine moderate	Morphine severe
3,618 ± 356	3,057 ± 157	2,857 ± 342	3,361 ± 543

Values are expressed as counts per minute bound per tube and are shown as means ± SEM from 9–11 separate experiments in each case, performed in triplicate or quadruplicate. The values were not significantly different, one-way ANOVA with Bonferroni post-test. The same amount of membrane protein (10 μg/tube) was used in each case

When tested at a concentration of 1 μM, GSK1521498 did not reduce basal [^35^S]GTPγS binding to brain membranes prepared from naïve, acute and moderate morphine-pretreated animals (Fig. 4). Only in membranes from severe morphine-pretreated animals did GSK1521498 show very slight but statistically significant inhibition of basal [^35^S]GTPγS binding (Fig. 4).

## Discussion

Using recombinant human opioid receptors, we observed GSK1521498 to be a selective MOPr antagonist with some 14-fold selectivity for MOPr over both DOPr and KOPr and 100-fold selectivity for MOPr over NOPr. This relative selectivity for MOPr, DOPr and KOPr is similar to that reported by Ignar et al. (2011), but in the present study, the absolute potency of GSK1521498 was some 10-fold higher at each receptor type than previously reported. Compared to naltrexone, GSK1521498 was more selective for MOPr and had greater affinity for MOPr binding. We also observed that whereas GSK1521498 could completely antagonize MOPr activation by an exogenous agonist challenge, naltrexone, naloxone and 6-β-naltrexol could achieve only about 70 % blockade even at high doses.

Positive and negative allosteric modulation of G protein-coupled receptors (GPCRs) has been widely reported (see Keov et al. (2011) for review). Novel allosteric modulators of MOPr have recently been described (Burford et al. 2013). GSK1521498 is structurally unrelated to other MOPr selective antagonists. It was therefore important to determine whether or not it acted at the same orthosteric binding site as classical antagonists that have chemical structures based on the morphine structure (Corbett et al. 2006). GSK1521498 displaced all [^3^H]naloxone-specific binding to MOPr and did not alter the rate of [^3^H]naloxone dissociation from MOPr. Both of these observations are compatible with it acting at the same orthosteric site as naloxone.

We and others (Ignar et al. 2011) have observed that GSK1521498 exhibits inverse agonist activity on highly overexpressed recombinant MOPr. Overexpression of receptors is likely to facilitate the measurement of MOPr constitutive activity thus revealing inverse agonist activity in drugs otherwise thought to be neutral antagonists. We did not, however, observe inverse agonist activity of GSK1521498 when the level of expression was lower either on recombinant MOPr in CHO cells or on endogenous MOPr in mouse brain tissue from control animals. In brain tissue from animals receiving morphine pre-treatment of increasing severity, there was a tendency for GSK1521498 to exhibit inverse agonist activity, but this was slight and only achieved statistical significance with the most severe morphine pre-treatment regimen. However, the slight inverse agonism of GSK1521498 at high levels of receptor overexpression, or the highest dose of morphine pre-treatment, was in contrast to the behaviour of the other MOPr ligands in this assay. We did not observe inverse agonist activity for β-CNA or naltrexone in brain tissue from mice receiving any of the morphine pre-treatments. This is in contrast to Wang et al. (2001, 2004) who have observed inverse agonism activity for these compounds on endogenous MOPr. In the studies of recombinant human MOPr overexpressed in cell lines, we confirmed prior data that naltrexone (Ignar et al. 2011) had partial agonist activity, as did naloxone and the putative neutral antagonist 6-β-naltrexol. Recently, Bourassa et al. (2014) have also reported that naloxone and naltrexone behave as partial agonists at MOPr.

Opioid pre-treatment has been reported to increase MOPr constitutive activity presumably by increasing the phosphorylation state of MOPr (Liu et al. 2001; Sally et al. 2010; Wang et al. 2004, 2007; Xu et al. 2007 but see Divin et al. 2009). We did not observe any increase in basal GTPγS binding in mouse brain homogenates following any of the morphine pre-treatments. In our brain membrane experiments, the assay buffer contained Na^+^ which may reduce the level of constitutive G-protein signalling (Liu et al. 2001; Szekeres and Traynor 1997), but we used conditions identical to those of Wang et al. (2004) who had previously observed MOPr constitutive activity. Selley et al. (1997) have also failed to observe any increase in MOPr constitutive activity following morphine pre-treatment. While the reasons for this discrepancy are unknown, there might be subtle methodological factors that may be relevant. For example, it has been pointed out that in chronic opioid-pre-treatment studies, it is essential to ensure that all of the pre-treating agonist has been removed from tissue before evaluating constitutive activity and potential inverse agonists (Williams et al. 2013). We conclude that the inverse agonist action of GSK1521498 is not simply revealed by an elevated level of MOPr constitutive activity following chronic morphine treatment but results from some other as yet unidentified modification of MOPr function.

Functional studies in neurons and cell lines under physiological conditions have failed to detect MOPr constitutive activity after chronic morphine exposure in locus coeruleus neurons (Connor et al. 1999), periaqueductal gray neurons (Bagley et al. 2005) or SH-SY5Y cells (Kennedy and Henderson 1992). Indeed, the ability of other GPCRs to exhibit constitutive activity can be cell context-dependent, with for example, group I mGluRs showing extensive constitutive activity in expression systems but not in neurons, the reason for the latter being that in brain the receptor interacts with Homer proteins that suppress constitutive activity (Ango et al. 2001). More recently, MOPr constitutive activity and inverse agonist activity of naloxone and naltrexone were observed in dorsal root ganglion neurons cultured from βarrestin2 knockout but not wild-type mice (Lam et al. 2011; Walwyn et al. 2007).

The issue of whether MOPrs exhibit constitutive activity and whether some antagonists are actually inverse agonists remains confusing with different results being observed by different groups using apparently similar experimental procedures (see Connor and Traynor 2010). The present results indicate that whilst GSK1521498 could exhibit inverse agonist activity under conditions where MOPr are highly overexpressed, but not at lower level of expression of recombinant receptors or at untreated endogenous MOPrs, the other ligands examined (naloxone, naltrexone and 6-β-naltrexol) did not show any inverse agonist activity but instead under conditions of high receptor expression, displayed partial agonist activity.

Finally, we return to the question of how these pharmacological differences between GSK1521498 and other MOPr ligands could be related to the previously reported behavioural differences between GSK1521498 and naltrexone (Giuliano et al. 2013). In this prior study, both GSK1521498 and naltrexone inhibited drug-seeking behaviour for cocaine or heroin in a second-order reinforcement paradigm, but this effect was significantly greater for GSK1521498 compared to an equivalent dose of naltrexone. Moreover, GSK1521498 inhibited further drug-seeking behaviour after a first self-administration of heroin, which naltrexone did not. There are many possible pharmacological reasons for the apparently greater efficacy of GSK152498 to modulate behaviour in these rodent models of drug dependence. As previously shown, and confirmed independently here, GSK1521498 is more selective for the mu opioid receptor, which is theoretically implicated in reward signalling. It is also conceivable that the more complete antagonism achieved by GSK1521498, and its slight inverse agonist activity under some assay conditions, compared to the incomplete antagonism and partial agonist activity of naltrexone (and the other opioid receptor ligands studied), might contribute to its different behavioural profile, by more completely attenuating MOPr-mediated signalling in brain centres controlling reward-driven behaviour. However, we note that the inverse agonist activity of GSK1521498 was only demonstrated under conditions of high receptor expression in vitro or in mice pre-treated with the highest dose of morphine. It is debatable to what extent these assay conditions are typical of normal physiological or pathophysiological states of the MOPr system.

## References

[CR1] Ango F, Prezeau L, Muller T, Tu JC, Xiao B, Worley PF, Pin JP, Bockaert J, Fagni L (2001). Agonist-independent activation of metabotropic glutamate receptors by the intracellular protein Homer. Nature.

[CR2] Bagley EE, Chieng CH, Chistie MJ, Connor M (2005). Opioid tolerance in periaqueductal gray neurons isolated from mice chronically treated with morphine. Br J Pharmacol.

[CR3] Bailey CP, Llorente J, Gabra BH, Smith FL, Dewey WL, Kelly E, Henderson G (2009). Role of protein kinase C and μ-opioid receptor (MOPr) desensitization in tolerance to morphine in rat locus coeruleus neurons. Eur J Neurosci.

[CR4] Bourassa P, Bagheri H, Pineyro G, Grandbois M, Gendron L (2014) Label-free monitoring of μ opioid receptor-mediated signaling. Mol Pharmacol 10.1124/mol.114.093450 24874699

[CR5] Brillet K, Kieffer BL, Massotte D (2003). Enhanced spontaneous activity of the μ opioid receptor by cysteine mutations: characterization of a tool for inverse agonist screening. BMC Pharmacol.

[CR6] Burford NT, Wang D, Sadée W (2000). G-protein coupling of μ-opioid receptors (OP3): elevated basal signaling activity. Biochem J.

[CR7] Burford NT, Clark MJ, Wehrman TS, Gerritz SW, Banks M, O'Connell J, Traynor JR, Alt A (2013). Discovery of positive allosteric modulators and silent allosteric modulators of the μ-opioid receptor. Proc Natl Acad Sci U S A.

[CR8] Connor M, Traynor J (2010). Constitutively active μ-opioid receptors. Methods Enzymol.

[CR9] Connor M, Borgland SL, Christie MJ (1999). Continued morphine modulation of calcium channel currents in acutely isolated locus coeruleus neurons from morphine-dependent rats. Br J Pharmacol.

[CR10] Corbett AD, Henderson G, McKnight AT, Paterson SJ (2006). 75 years of opioid research: the exciting but vain quest for the Holy Grail. Br J Pharmacol.

[CR11] Divin MF, Bradbury FA, Carroll FI, Traynor JR (2009). Neutral antagonist activity of naltrexone and 6β-naltrexol in naïve and opioid-dependent C6 cells expressing a μ-opioid receptor. Br J Pharmacol.

[CR12] Giuliano C, Robbins TW, Nathan PJ, Bullmore ET, Everitt BJ (2012). Inhibition of opioid transmission at the μ-opioid receptor prevents both food seeking and binge-like eating. Neuropsychopharmacology.

[CR13] Giuliano C, Robbins TW, Wille DR, Bullmore ET, Everitt BJ (2013). Attenuation of cocaine and heroin seeking by μ-opioid receptor antagonism. Psychopharmacology (Berl).

[CR14] Ignar DM, Goetz AS, Noble KN, Carballo LH, Stroup AE, Fisher JC, Boucheron JA, Brainard TA, Larkin AL, Epperly AH, Shearer TW, Sorensen SD, Speake JD, Hommel JD (2011). Regulation of ingestive behaviors in the rat by GSK1521498, a novel μ-opioid receptor-selective inverse agonist. J Pharmacol Exp Ther.

[CR15] Kennedy C, Henderson G (1992) Chronic exposure to morphine does not induce dependence at the level of the calcium channel current in human SH-SY5Y cells. Neuroscience 49:937–944 10.1016/0306-4522(92)90369-d 1279457

[CR16] Keov P, Sexton PM, Christopoulos A (2011). Allosteric modulation of G protein-coupled receptors: a pharmacological perspective. Neuropharmacology.

[CR17] Lam H, Maga M, Pradhan A, Evans CJ, Maidment NT, Hales TG, Walwyn W (2011). Analgesic tone conferred by constitutively active μ opioid receptors in mice lacking β-arrestin 2. Mol Pain.

[CR18] Liu JG, Prather PL (2001). Chronic exposure to μ-opioid agonists produces constitutive activation of μ-opioid receptors in direct proportion to the efficacy of the agonist used for pretreatment. Mol Pharmacol.

[CR19] Liu JG, Ruckle MB, Prather PL (2001). Constitutively active μ-opioid receptors inhibit adenylyl cylcase activity in intact cells and activated G-proteins differently than the agonist [D-Alal^2^N-MePhe^4^Gly-ol^5^]enkephalin. J Biol Chem.

[CR20] Nathan PJ, O'Neill BV, Bush MA, Koch A, Tao WX, Maltby K, Napolitano A, Brooke AC, Skeggs AL, Herman CS, Larkin AL, Ignar DM, Richards DB, Williams PM, Bullmore ET (2012). Opioid receptor modulation of hedonic taste preference and food intake: a single-dose safety pharmacokinetic and pharmacodynamic investigation with GSK1521498, a novel μ-opioid receptor inverse agonist. J Clin Pharmacol.

[CR21] Nathan PJ, Bush MA, Tao WX, Koch A, Davies KM, Maltby K, O'Neill BV, Napolitano A, Skeggs AL, Brooke AC, Richards DB, Williams PM, Bullmore ET (2012). Multiple-dose safety pharmacokinetics and pharmacodynamics of the μ-opioid receptor inverse agonist GSK1521498. J Clin Pharmacol.

[CR22] Patrick GA, Dewey WL, Spaulding TC, Harris LS (1975). Relationship of brain morphine levels to analgesic activity in acutely treated mice and rats and in pellet implanted mice. J Pharmacol Exp Ther.

[CR23] Raehal KM, Lowery JJ, Bhamidipati CM, Paolino RM, Blair JR, Wang D, Sadée W, Bilsky EJ (2005). In vivo characterization of 6beta-naltrexol an opioid ligand with less inverse agonist activity compared with naltrexone and naloxone in opioid-dependent mice. J Pharmacol Exp Ther.

[CR24] Sally EJ, Xu H, Dersch CM, Hsin LW, Chang LT, Prisinzano TE, Simpson DS, Giuvelis D, Rice KC, Jacobson AE, Cheng K, Bilsky EJ, Rothman RB (2010). Identification of a novel ‘almost neutral’ μ-opioid receptor antagonist in CHO cells expressing the cloned human μ-opioid receptor. Synapse.

[CR25] Selley DE, Nestler EJ, Breivogel CS, Childers SR (1997). Opioid receptor-coupled G-proteins in rat locus coeruleus membranes: decrease in activity after chronic morphine treatment. Brain Res.

[CR26] Szekeres PG, Traynor JR (1997). Delta opioid modulation of the binding of guanosine-5′-O-(3-[^35^S]thio)triphosphate to NG108-15 cell membranes: characterization of agonist and inverse agonist effects. J Pharmacol Exp Ther.

[CR27] Walwyn W, Evans CJ, Hales TG (2007). β-arrestin2 and c-Src regulate the constitutive activity and recycling of μ opioid receptors in dorsal root ganglion neurons. J Neurosci.

[CR28] Wang D, Raehal KM, Bilsky EJ, Sadée W (2001). Inverse agonists and neutral antagonists at μ-opioid receptor (MOPr): possible role of basal receptor signaling in narcotic dependence. J Neurochem.

[CR29] Wang D, Raehal KM, Lin ET, Lowery JJ, Kieffer BL, Bilsky EJ, Sadée W (2004). Basal signaling activity of μ opioid receptor in mouse brain: role in narcotic dependence. J Pharmacol Exp Ther.

[CR30] Wang D, Sun X, Sadee W (2007). Different effects of opioid antagonists on μ− δ− and κ-opioid receptors with and without agonist pretreatment. J Pharmacol Exp Ther.

[CR31] Williams JT, Ingram SL, Henderson G, Chavkin C, von Zastrow M, Schultz S, Koch T, Evans C, Christie MJ (2013). Regulation of μ-opioid receptors: desensitization phosphorylation internalization and tolerance. Pharmacol Rev.

[CR32] Xu H, Partilla JS, Wang X, Rutherford JM, Tidgewell K, Prisinzano TE, Bohn LM, Rothman RB (2007). A comparison of noninternalizing (herkinorin) and internalizing (DAMGO) μ-opioid agonists on cellular markers related to opioid tolerance and dependence. Synapse.

